# HIV-1 Natural Antisense Transcription and Its Role in Viral Persistence

**DOI:** 10.3390/v13050795

**Published:** 2021-04-29

**Authors:** Rui Li, Rachel Sklutuis, Jennifer L. Groebner, Fabio Romerio

**Affiliations:** 1 Department of Molecular and Comparative Pathobiology, Johns Hopkins University School of Medicine, Baltimore, MD 21205, USA; rli74@jhmi.edu; 2 HIV Dynamics and Replication Program, Host-Virus Interaction Branch, National Cancer Institute, National Institutes of Health, Frederick, MD 21702, USA; rachel.sklutuis@nih.gov (R.S.); jenn.groebner@nih.gov (J.L.G.)

**Keywords:** HIV-1, natural antisense transcription, long non-coding RNA, expression, latency, persistence, epigenetic silencing

## Abstract

Natural antisense transcripts (NATs) represent a class of RNA molecules that are transcribed from the opposite strand of a protein-coding gene, and that have the ability to regulate the expression of their cognate protein-coding gene via multiple mechanisms. NATs have been described in many prokaryotic and eukaryotic systems, as well as in the viruses that infect them. The human immunodeficiency virus (HIV-1) is no exception, and produces one or more NAT from a promoter within the 3’ long terminal repeat. HIV-1 antisense transcripts have been the focus of several studies spanning over 30 years. However, a complete appreciation of the role that these transcripts play in the virus lifecycle is still lacking. In this review, we cover the current knowledge about HIV-1 NATs, discuss some of the questions that are still open and identify possible areas of future research.

## 1. Introduction

Until recently, RNA was thought to be a mere messenger, transferring instructions from a DNA depositary of the genetic information to proteins that regulate biological processes. Thus, the presence in higher eukaryotes of very large genomic regions that do not encode proteins or that do not act as *cis*-acting regulatory elements (i.e., introns and intergenic sequences) were simply considered to be ‘junk DNA’. However, in recent years, it has become increasingly evident that this view was incorrect. Indeed, international research consortia such as the FANTOM, GENCODE and ENCODE projects showed that up to 90% of eukaryotic genomes are transcribed into RNA [1]. However, only 1%–2% of the transcripts carry information that is translated into proteins, whereas the vast majority of RNA molecules are non-protein-coding. Furthermore, although it has long been known that some non-coding RNAs (ncRNA) such as rRNAs, tRNAs, snRNAs and snoRNAs serve generic housekeeping functions within the cell, others have biological functions that include gene regulation.

## 2. Non-Coding RNAs

### 2.1. Phylogenetic Distribution and Complexity of ncRNAs

The expression of ncRNAs has been documented across the evolutionary spectrum. Indeed, ncRNAs have been identified in *Escherichia coli* and other bacteria [2,3,4,5,6], in archaea [7] and bacteriophages [8]. In these organisms, ncRNAs primarily play a role in regulating mRNA translation. However, prokaryotic genomes mostly contain protein-coding sequences that are highly variable in terms of their repertoire, even between closely related strains [9,10], and the number of regulatory proteins increases exponentially with genome size [11]. Relying on a protein-based regulatory system has limited the evolution and complexity of prokaryotes [9,12,13]. Thus, speciation and evolution cannot be easily reconciled with the restrictions of protein-coding genomes [14].

In contrast, eukaryotes present a relatively stable proteome, both in terms of repertoire and sequence conservation. Despite significantly different developmental and physiological complexities, the genomes of very different organisms such as *Caenorhabditis elegans* (10^3^ cells) and *Homo sapiens* (10^14^ cells) contain a number of protein-coding genes that vary by less than 30%. Although the proportion of the eukaryotic genome occupied by protein-coding genes declines with increasing organismal complexity, the fraction of transcribed non-protein-coding sequences increases [15,16]. Eukaryotes developed a regulatory system based on RNA, along with the protein infrastructure needed to recognize and employ this system [9]. In humans, at least 70% of the genome is transcribed on one or both strands [17], but only 2% of transcripts are protein-coding [18]. Thus, a progressive shift from protein-coding to non-coding RNAs occurred along the evolutionary scale, suggesting that the repertoire of regulatory ncRNAs had an impact on evolution and speciation [9,16,19,20,21].

### 2.2. Classification of ncRNAs

Non-coding RNAs (ncRNA) encompass a very heterogenous group of molecules that includes ribosomal (rRNA), transfer (tRNA), small nuclear (snRNA), small nucleolar (snoRNA), Piwi-interacting (piRNA), micro (miRNA) and long non-coding RNA (lncRNA) [22]. ncRNAs can be classified based on their size, and the arbitrary length of 200 nt is used to discriminate short vs. long ncRNAs. Alternatively, ncRNAs can be classified based on their housekeeping vs. regulatory role [23]. However, neither method of classification is perfect.

LncRNAs represent the most abundant group of non-coding transcripts [24]. These are defined as molecules of >200 nt in length with a primarily regulatory function. Most lncRNAs are characterized by predominantly nuclear localization, low expression levels, and can be either polyadenylated or not [22,25,26]. Although lncRNAs show a lower degree of genetic sequence and gene structure conservation compared to protein-coding genes [27,28,29], this does not imply a lack of function [30,31]. Indeed, several examples suggest that lncRNAs follow different patterns of conservation underlying their function compared to protein-coding mRNAs, which must maintain an open reading frame. There is evidence that the conservation of secondary structure and functional modules—rather than sequence—underlies the biological activity of lncRNAs [32,33,34]. Secondary structure may be responsible for the functional interaction between lncRNAs and proteins, RNA, DNA and chromatin [34]. For instance, many lncRNAs interact with polycomb proteins despite very low sequence similarity [35].

LncRNAs can be classified into five categories based on their relative proximity to protein-coding genes [32,36]: (i) intergenic transcripts (when there is no overlap with or proximity to a protein-coding gene; these are known as long intergenic non-coding RNA or lincRNA); (ii) bidirectional transcripts (when the expression of a protein-coding and a non-coding gene on opposite strands is initiated in close genomic proximity); (iii) intronic transcripts (when the lncRNA is derived entirely from within the intron of a second transcript); (iv) sense transcripts (when the lncRNA overlaps and is transcribed in the same orientation as that of a protein-coding gene); and (v) antisense transcripts (when the lncRNA overlaps and is transcribed in the opposite orientation to that of a protein-coding gene; these are known as natural antisense transcripts or NATs).

## 3. Natural Antisense Transcripts (NATs)

### 3.1. Phylogenetic Distribution and Conservation

Natural antisense transcripts (NATs) are a class of long non-coding RNAs, first described in bacteria and bacteriophages [37,38,39]. Later, examples also emerged in eukaryotic systems, including yeast, invertebrates and chordates [40,41,42,43]. Over the last 20 years, genome-wide analyses showed that antisense transcription is a widespread phenomenon in all kingdoms of life [44,45,46,47]. In higher eukaryotes, 20%–40% of protein-coding genes (especially those with tissue-specific expression) contain antisense transcription [27,48,49,50,51]. In mammals, developing sperm cells show the highest level of antisense transcription [52]. On the contrary, *Caenorhabditis elegans* shows a significantly lower number of NATs [53].

As discussed above for the broader class of lncRNAs, the genetic and primary sequence of antisense transcripts is poorly conserved across species [28,54,55,56,57], but it has been proposed that their secondary and tertiary structures are conserved and essential for their function [34,58].

### 3.2. Characteristics of NATs Expression

On average, NATs are expressed at a >10-fold lower abundance and have fewer splicing events than sense transcripts [59,60]. They show preferential nuclear accumulation [61] and low fidelity of transcription initiation [62] and can originate from independent, bidirectional or cryptic promoters [58].

**Figure 1:**
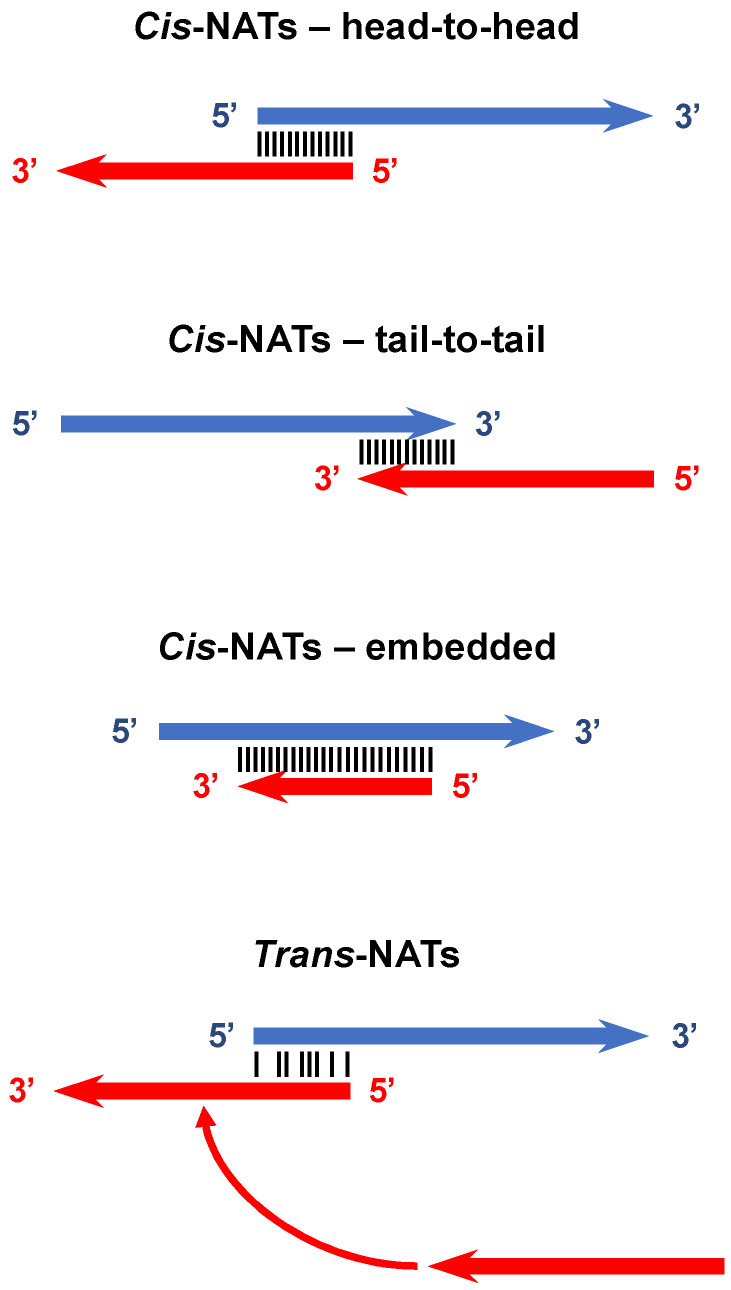
Types of natural antisense transcripts and overlaps with sense transcripts. NATs are divided into *cis* and *trans*. The former is expressed from the same genetic locus as their paired sense transcript. They can be organized in a head-to-head (overlap of the 5′ ends), tail-to-tail (overlap of the 3′ ends) or fully embedded (complete overlap) fashion. *Trans*-NATs are expressed from a different genetic locus and share only partial homology with their paired sense transcript.

Antisense transcripts can be subdivided into *cis*-NATs and *trans*-NATs (Figure 1) [63]. In the first case, sense and antisense transcripts originate from the same genomic *locus*, and they can be organized in a head-to-head (divergent expression), tail-to-tail (convergent expression) or fully embedded fashion (internal expression) [50,58]. Thus, in the area of overlap, sense and antisense transcripts share complete complementarity. On the contrary, *trans*-NATs originate from a different genomic region of their paired sense transcript; therefore, in the area of overlap, the two transcripts are only partially complementary [63]. The expression of sense–antisense pairs is coordinated (either co-expressed or inversely expressed) more frequently than expected by chance, especially in tail-to-tail pairs, suggesting a functional relevance [64,65]. Indeed, NATs have the ability to regulate the expression of their paired sense transcript in a highly locus-specific manner [23]. Generally, NATs lack open reading frames (ORFs), but cases of NATs with protein-coding potential have been reported [66,67,68]. Thus, while NATs initially may not encode proteins, they provide a substrate on which evolution gives rise to open reading frames [58].

### 3.3. Mechanisms of Gene Regulation by NATs

A number of studies have shown that NATs affect the expression of their cognate protein-coding sense genes [69]. In most cases, NATs exert a negative regulatory function on the protein-coding gene, although there are reports showing that some NATs can protect their paired sense transcript from nuclease degradation [70]. The function of antisense transcripts is mediated by the RNA molecule itself and/or by the act of antisense transcription itself. This can occur at multiple levels—transcription initiation, RNA processing, RNA transport, RNA stability and translation [50]. Some mechanisms require co-expression of sense and antisense RNA; others require mutual exclusion [50]. Below, we briefly describe the mechanisms through which NATs can regulate expression of their paired sense transcript. For comprehensive reviews, see [58,71,72].

At the level of transcription initiation, NATs can inhibit the expression of the sense gene through at least four mechanisms, collectively known as transcriptional interference (TI) [72]. The first one is promoter competition, which occurs when sense and antisense RNA are expressed from a bidirectional promoter. In this case, the assembly of the transcriptional machinery expressing the antisense RNA blocks or prevents the formation of the transcriptional machinery expressing the sense RNA. The second mechanism is binding site occlusion, in which the passage of the RNA polymerase complex expressing the NAT blocks access to the chromatin of transcription factors required for expression of the sense transcript. The third mechanism is RNA polymerase collision. This occurs when an elongating transcriptional machinery displaces another already assembled onto its promoter (‘sitting duck’), or vice versa when an RNA polymerase complex stalls an incoming elongating transcriptional complex (‘roadblock’). The fourth mechanism involves the orchestration of DNA and chromatin changes by the antisense transcript, resulting in epigenetic silencing of the sense gene. This mechanism is the most frequent in the case of sense–antisense transcripts showing discordant expression [23], and is based on the ability of NATs to form flexible modular scaffolds in which different domains of the NAT molecule can interact with DNA and proteins to generate specific functional complexes. The modular structure of NATs allows them to act as regulatory hubs, which direct DNA and chromatin-modifying complexes that either lack DNA-binding capacity or DNA sequence specificity to a specific genomic location. Thus, the tethering of DNA- and chromatin-modifying enzymes by NATs results in epigenetic silencing of sense gene expression [23,58]. For instance, NATs tether or guide the polycomb repressor complex 2 (PRC2) to target genes, leading to the di- and trimethylation of lysine 27 on histone H3 (H3K27me2/3), nucleosome assembly and transcriptional silencing. The mechanism of action involving epigenetic silencing appears to be the most frequent, and it explains functionality even in the case of the low abundance of NATs, because the genomic sequence targeted by each NAT is present in only two copies per nucleus.

At the post-transcriptional level, NATs can regulate the expression of their paired sense transcript via the formation of double-stranded RNA complexes in at least four ways. The first one—termed RNA masking—involves the formation of a sense–antisense duplex that blocks the interaction of the sense transcript with factors (proteins and miRNAs) that regulate its splicing, stability, transport and translation [71,72]. The second mechanism described is called RNA interference, and it entails the recognition of the RNA duplex by Dicer, with subsequent cleavage and formation of ‘endo-siRNAs’ [72]. The third mechanism is based on intracellular immune responses triggered by dsRNA molecules typical of viral pathogens. These molecules are recognized by protein kinase R (PKR), which undergoes dimerization and autophosphorylation, suppresses protein expression and ultimately triggers IFNα/β innate immune responses. Finally, dsRNA molecules can be recognized by members of the ADAR protein family, which deaminate adenosine residues into inosine in a process called RNA editing. Since inosine pairs more efficiently with cytosine than thymidine, this may result in amino acid changes [71].

### 3.4. Protein-Based vs. NAT-Based Gene Regulation

Systems in which NATs regulate sense gene expression at multiple levels (e.g., transcriptional and post-transcriptional) achieve more efficient (less noisy) gene repression than transcription factor-based systems, because any transcriptional leakage is blocked post-transcriptionally [58]. If expression of the NAT precedes that of the sense transcript, then the NAT acts a buffer. It sets a threshold, dampening stochastic variations in the expression of sense RNA, which can be expressed only when it exceeds such threshold. Thus, higher NAT levels increase the threshold to be overcome before the sense transcript can achieve maximal expression. In this case, sense–antisense pairs are self-regulatory circuits that exist in an ‘on’ (sense RNA expressed, antisense RNA not expressed) or ‘off’ state (sense RNA not expressed, antisense RNA expressed) [50,58]. The presence of the antisense transcript establishes an ultrasensitive, threshold-dependent on–off switch for sense-gene regulation. In this system, the activating stimulus driving sense gene expression has to be high enough to oppose the repressive/buffering effect of the antisense transcript before the equilibrium can be altered (triggering the on–off switch) and an increase in sense gene expression can be achieved. Moreover, upon removal of the activating stimulus, expression of the sense gene returns to the basal level more rapidly [73]. Examples can be found in *Synechocistic* spp. cyanobacteria, in *Saccharomyces cerevisiae* and in human T cell leukemia virus type 1 (HTLV-1) [74,75,76,77].

### 3.5. Examples of NATs in Eukaryotic Cell Systems

In eukaryotes, NATs use a variety of mechanisms (as described above) to regulate gene expression in physiological processes (Table 1). In some cases, alterations in NAT expression have also been associated with human diseases such as cancers, neurodegenerative and cardiovascular diseases [78]. One of the most common mechanisms used by antisense transcripts to control the transcription of sense genes is the recruitment of chromatin-remodeling complexes to the DNA to induce histone modifications and DNA methylation [58]. For example, X chromosome inactivation (XCI) involves transcriptional regulation of X-inactive specific transcript (*Xist*) by an antisense transcript (*Tsix),* both of which are located within the X inactivation center *(Xic)* on the X chromosome. On the inactive X chromosome, *Xist* is expressed and coats the chromosome. In this way, *Xist* acts as a scaffold in the recruitment of PRC2 proteins that induce trimethylation of lysine 27 on histone H3 (H3K27me3), leading to the formation of heterochromatin [79,80]. On the active X chromosome in mice, the expression of the antisense transcript *Tsix* interferes with the sense transcription of *Xist* through the recruitment of DNA methyltransferases that induce methylation of the Xist promoter, thereby inhibiting the expression of *Xist* and preventing the inactivation of genes. [81,82,83].

Although X-inactivation affects multiple loci across the whole chromosome, transcriptional silencing mediated by NATs also occurs at specific domains, such as in *ANRIL*-induced silencing of the INK4 locus (also known as the CDKN2B-CDKN2A locus). The INK4 locus encodes the p15, p14 and p17 tumor suppressor genes, which function in cell proliferation, apoptosis, senescence and aging [84]. Multiple short and long isoforms of *ANRIL* exist, and single-nucleotide polymorphisms within *ANRIL* have been implicated in the development of cardiovascular disease, cancer, diabetes, glaucoma and endometriosis [84,85]. *ANRIL* acts to silence the INK4 locus through the recruitment of the PRC1 and PRC2 complexes to induce histone modifications. Specifically, *ANRIL* recruits PRC2 to catalyze the methylation of H3 lysine 27 (H3K27) by binding to SUZ12, a component of the complex. *ANRIL* also binds chromobox 7 (CBX7), a PRC1 protein that recognizes histone methylated lysine (H3K27), and catalyzes mono-ubiquitination of histone 2A (H2A-K119) to signal for the continued repression of the locus [86,87]. Additionally, PRC1 and PRC2 proteins (CBX7 and EZH2) interact with DNA methyltransferase 3b (Dnmt3b) to induce promoter methylation and further silencing of genes in the locus [84].

Silencing of imprinted domains is also regulated by NATs. Potassium voltage-gated channel subfamily q member 1 opposite strand/antisense transcript 1 (*Kcnq1ot1*) regulates the expression of *Kcnq1* loci of the paternal allele [88]. In the embryo, genes located in the central portion of the domain are silenced, whereas in the placenta, more than 10 genes, including those proximal and distal to the central region, are also silenced [89,90]. Briefly, the Kcnq1ot1 promoter at the KvDMR1 imprinting control region (ICR) is unmethylated on the paternal allele but is methylated on the maternal allele. Therefore, *Kcnq1ot1* is expressed from the paternal allele and spreads in a bidirectional cloud, resulting in the silencing of several genes in the locus, promoting heterochromatin formation [91,92]. CCCTC-binding factor (CTCF), a transcriptional regulator, binds to sites at the KvDMR1 ICR of the paternal allele and prevents the spreading of CpG methylation and the silencing of antisense transcript expression by the surrounding heterochromatin [100]. *Kcnq1ot1* associates with EED, a component of the PRC2 complex, to recruit other PRC2 complex proteins, including EZH2 and histone methyltransferases G9a/EHMT2, which catalyze histone 3 lysine 27 trimethylation (H3K27me3) and histone 3 lysine 9 bi/trimethylation (H3K9me2/3), respectively [88,89]. Imprinting mechanisms may be differentially regulated. For example, in somatic tissues, DNA methyltransferase 1 (Dnmt1) is thought to maintain methylation at the *Kcnq1* locus but PRC2-induced histone methyltransferases may play more of a role in placental tissues [89,91,101].

NATs can also regulate sense transcription after initiation has occurred. In *S. cerevisiae*, the antisense transcript, regulator of meiosis 2 (*RME2*), blocks elongation of the full-length inducer of meiosis 4 (*IME4*) sense transcript, but not its initiation in diploid cells. *RME2*-mediated repression requires the presence of a specific 450-bp region (residues 225 to 675) within *IME4* [93,94]. Although the detailed mechanism is still unclear, it has been speculated that chromatin remodeling/modifying enzymes may be recruited to this region or that this region may be sensitive to bi-directional transcription, thereby allowing transcription in one direction and interrupting transcription on the opposite strand [93]. During starvation conditions in diploid cells, *RME2* antisense transcription is blocked by the α1-α2 repressor binding downstream of *IME4*, which allows transcription initiation and elongation of the full-length *IME4* sense transcript to proceed and signals the switch from mitotic to meiotic division [93,94].

At the post transcriptional level, antisense transcripts β-site APP-cleaving enzyme gene 1 (*BACE1-AS*) and zinc-finger E-box-binding homeobox 2 gene (*ZEB2-AS*) use RNA masking to regulate gene expression [58]. BACE1 encodes the β-secretase enzyme, which has been shown to be a driver of Alzheimer’s disease-associated pathology. *BACE1-AS* binds BACE1 to form a duplex that masks the microRNA miR485-5p binding site and prevents miRNA-mediated degradation of mRNA and translational repression of BACE1 [69,95]. The BACE1 protein plays an essential role in cognitive, emotional and synaptic functions; however, dysregulation of protein levels leads to higher levels of amyloid-β 1-42 that form the β-amyloid plaques in the brain, which are characteristic of Alzheimer’s disease [96].

RNA masking can also regulate expression of transcript isoforms. In humans, *ZEB2* is a transcriptional repressor of E-cadherin [78] and contains an internal ribosome entry site (IRES) in the 5′ intron. The antisense transcript, *ZEB2-AS*, prevents the splicing of this intron, resulting in the transcription of an alternate isoform of the *ZEB2* mRNA. This alternate isoform retains the IRES and has increased translational efficiency when compared to the spliced isoform without the IRES. Alterations in regulatory mechanisms resulting in the overexpression of *ZEB2-AS* have been associated with acute myeloid leukemia [97], bladder cancer [98] and other cancers [99].

NATs can act as regulatory hubs by linking the ‘on’ and ‘off’ state of divergent neighboring genes [58]. In budding yeast, transcription of *SUR7*, which encodes a plasma membrane protein important for membrane organization and cell wall synthesis, is linked through a bidirectional promoter to GAL80, a transcriptional regulator in galactose metabolism. In the presence of galactose, transcription factors activate a bidirectional promoter that initiates the transcription of *GAL80* and *SUT719*, the upstream *SUR7* antisense transcript. *SUT719* represses the activation of the *SUR7* promoter and blocks the transcription of *SUR7* [58,76]. This transcriptional block by *SUT719* can be overcome if the activating signals on the *SUR7* promoter are strong enough to reach a certain threshold [76]. In this way, sense and antisense transcription induced by a bidirectional promoter act as a regulatory circuit, controlling multiple processes in yeast. Although these examples describe how NATs regulate transcription in eukaryotic cells, importantly, similar mechanisms are also employed by NATs encoded from genes in eukaryotic viruses.

### 3.6. Examples of NATs in Viral Systems

Natural antisense transcription has been documented in many eukaryotic viruses and below we discuss a few examples and their contributions to the virus lifecycle and pathogenesis (Table 2).

Roizman and colleagues showed that genes in the antisense direction to known herpesvirus genes are common [102], which is consistent with the observation that many individual NATs can be identified within the genomes of herpesvirus family members [102,103,104,105,106,107,108,109,110]. Herpesviruses are double-stranded DNA viruses that encode hundreds of viral proteins and have a viral lifecycle that is divided into latent and lytic stages. During lytic infection, many viral genes are expressed at high levels, and the infectious virus is actively produced. Immediate early genes are expressed first, and regulate the expression of subsequently expressed early and late genes. In contrast, no infectious virus is detected and the lytic gene expression program is shut down during the latent stage. The only abundant viral RNAs expressed are latency-associated transcripts (LATs), which are considered a hallmark of HSV-1 latency. LATs are antisense to the HSV immediate-early gene ICP0, a viral transactivator of lytic gene expression [111,112,113]. LATs were important in limiting the accumulation of viral lytic gene transcripts during the establishment and maintenance of latency in a mouse model, and have also been implicated in the epigenetic regulation of HSV gene expression via heterochromatinization of the lytic gene promoters [114,115,116,117,118]. Moreover, LATs may promote latency reactivation partly by inhibiting apoptosis and by promoting cell survival both in vitro and in vivo [119,120,121,122]. For instance, stable expression of LAT reduced the activation of the intrinsic pathway of apoptosis by inhibiting AKT dephosphorylation, and reducing activation of proapoptotic caspases [120,123]. LAT has also been shown to regulate apoptosis via downregulation of the JAK-STAT pathway during HSV-1 latency [148]. Similarly, Daniel and colleagues identified a unique spliced varicella zoster virus (VZV) latency-associated transcript (*VLT*) that lies antisense to the ICP0 homologue protein ORF61, and they showed that *VLT* specifically suppresses the expression of ORF61 in transfected cells [124]. Thus, VZV and HSV-1 have likely evolved a similar mechanism to regulate latency.

Antisense transcripts have also been identified in a variety of other viruses. Vladimir and colleagues identified several antisense transcripts in Epstein–Barr virus (EBV)-positive cells using genome-wide polyadenylation sequencing analysis [149]. Previous work also identified a group of latency-associated spliced transcripts that are antisense to the ICP4 homolog gene in Marek’s disease virus (MDV), including MDV small RNAs (MSRs) and 10-kb RNA [150]. Additionally, others reported that Kaposi’s sarcoma-associated herpesvirus (KSHV) encodes a lncRNA, named antisense-to-latency transcript (*ALT*), determined using a genome-tiling microarray [151]. A subsequent study confirmed that *ALT* is the NAT of the latency-associated nuclear antigen gene (LANA) and may play a role in regulating the viral lifecycle through a mechanism that remains to be elucidated [147]. Moreover, there are two other KSHV lncRNAs, T3.0 and T1.2, that are antisense to the mRNA of ORF50, although they have not been shown to affect its expression [152]. As more viral NATs are identified by next-generation sequencing technology, their role in the viral lifecycle will need to be elucidated.

Previous studies have also demonstrated the expression of antisense transcripts in human and animal retroviruses, such as bovine leukemia virus (BLV) [153], simian T-leukemia virus type 1 (STLV-1) [154], murine leukemia virus (MLV) [155], and bovine and feline immunodeficiency viruses (BIV and FIV) [156,157]. BLV and STLV-1 are viruses closely related to HTLV-1 (discussed below) that infect cows and non-human primates, respectively. BLV has been shown to express antisense transcripts in BLV-infected cell lines and PBMCs from asymptomatic BLV-infected animals [153]. Spliced antisense transcripts have been detected in Japanese macaques naturally infected with STLV-1, and these transcripts were found to function similarly to their counterparts in HTLV-1 [154]. Finally, MLV initiates transcription in the U3 region of the 3′ LTR to produce antisense transcripts (asU3). Integration of MLV within the host Jpd2 and Bach2 genes in the opposite orientation to their sense of transcription has been shown to give rise to chimeric asU3-Jpd2 and asU3-Bach2 transcripts, suggesting that asU3 may affect host gene expression [155].

One of the best characterized examples of a viral encoded NAT comes from studies of the human T cell leukemia virus 1 (HTLV-1). HTLV-1 is a retrovirus that predominantly infects CD4+ T cells. In 5%–10% of individuals living with HTLV-1, the virus can lead to an aggressive malignancy of T lymphocytes called adult T cell leukemia-lymphoma (ATL) or chronic inflammatory diseases known as HTLV-1-associated myelopathy (HAM) [125]. Antisense transcription in the HTLV-1 genome was first discovered in 1989 using the Northern blot analysis of RNA from an HTLV-1 infected cell line [126]. Expression of the HTLV-1 antisense transcript *Hbz* is initiated at several positions within the R and U5 regions of the proviral 3′LTR, which lacks a TATA box [127], and relies on Sp1, JunD, TCF1 and LEF1 promoter elements [128,129,130]. HTLV-1 antisense transcription is also regulated at the epigenetic level. Indeed, although the 3′LTR is rarely methylated [131,132,133,134], histone marks such as H3K9ac and H3K4me3 are highly enriched in the 3′LTR [135,136]. HTLV-1 produces three major *Hbz* RNA isoforms—an unspliced and two alternatively spliced (SP1 and SP2) transcripts [127,137]. In ATL cells, the expression levels of SP1 are higher than those of the unspliced form [138,139], but the two transcripts exhibit similar functions [140]. There is strong evidence that the *hbz* gene plays a role in the leukemic process following HTLV-1 infection. In about half of the ATL cases, the viral transactivator *tax* gene is not expressed due to deletion of the 5′LTR [141], epigenetic silencing of the 5′LTR [142] or mutations within the *tax* gene [143]. On the contrary, the 3′LTR and the pX region of HTLV-1 (which contains *hbz*) remain intact and *hbz* is transcribed in all ATL cases [144]. Although *Hbz* RNAs encode for a protein called HBZ, mutation of ATG does not affect the ability of the transcripts to promote cell proliferation and to inhibit apoptosis [144,145]. Indeed, expression of *Hbz* RNA is associated with induction of host genes involved in cell cycle progression and proliferation, and with the anti-apoptosis factor survivin [145]. In contrast, the HBZ protein promotes proviral latency via its interaction with several host factors that bind the viral cyclic AMP response elements (vCRE) in the HTLV-1 5′LTR, such as CREB, CREM and ATF-1 [77], thus precluding recruitment of the HTLV-1 transactivator, TAX, to the 5′LTR [146]. Additionally, HBZ has been shown to prevent the binding of TAX to the host factor CBP/p300, and its recruitment of the 5′LTR [77]. Altogether, there is strong evidence that both the RNA and protein products of the HTLV-1 antisense gene *hbz* play a role in leukemogenesis.

## 4. Natural Antisense Transcription in the HIV-1 Proviral Genome

### 4.1. Discovery of HIV-1 Natural Antisense Transcription

**Figure 2:**
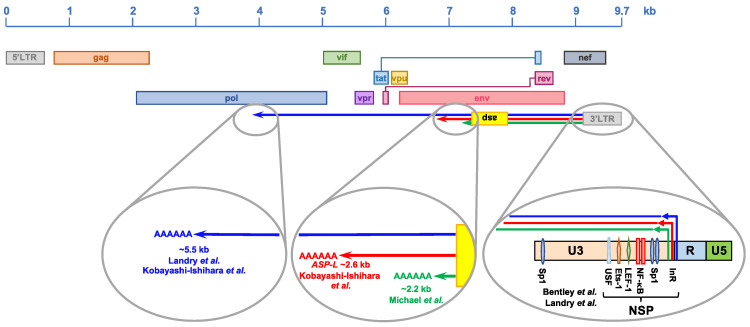
Schematic representation of HIV-1 NATs reported in the literature. The expression of HIV-1 NATs is dependent on a negative sense promoter (NSP) located in the U3 region of the 3′LTR. NSP is a TATA-less promoter that functions independently of Tat, and which relies on several housekeeping and inducible transcription factors, such as Sp1, NF-κB, LEF-1, Ets-1 and USF. The start site(s) of HIV-1 NATs are determined by initiator elements (InR) located in proximity to the U3-R boundary. Different research groups have described several NATs associated with the HIV-1 proviral genome. Their lengths vary between 2.2 and 5.5 kb, they all encompass the ORF encoding for the HIV-1 antisense protein (ASP) and they are all polyadenylated.

The existence of an antisense gene within the HIV-1 proviral genome was first proposed in 1988 by Roger Miller, who identified a highly conserved ORF in the minus strand of twelve viral isolates [158]. The antisense gene was found in the −2 reading frame, and maps in the same genomic region as the *env* gene, straddling the gp120/gp41 boundary (Figure 2) [158]. It was predicted to encode for a protein of ~190 amino acids with an unusually high content of hydrophobic residues, which suggested a possible association with cellular membranes [158]. In addition to the length of the ORF (>100 codons, which is uncommon in DNA strands complementary to known genes [159]), two lines of evidence were proposed in support of the existence of the antisense gene—first, the presence of regulatory sequences both at the 5′ and 3′ ends of the ORF, which are required for RNA expression and processing [158], and second, a codon periodicity of ‘G-nonG-N’ nucleotides that is more typical of protein-coding than non-protein coding genes [158].

Although the seminal study on the HIV-1 antisense gene did not provide experimental evidence for antisense transcription in the HIV-1 genome, this was demonstrated shortly thereafter, through the use of Northern blot analysis of poly-A+ RNA extracted from H9 cells acutely infected with HIV-1 strain IIIB [160]. Interestingly, this report showed that antisense transcription was restricted to the early phases of acute infection, and produced three transcripts of 1.6, 1.1 and 1.0 kb [160]. Antisense transcription could not be detected through Northern blot analysis during late-phase acute infection [160] or chronic infection [161]. However, the use of RT-PCR confirmed the expression of antisense transcripts in the HIV-1 proviral genome in acutely-infected H9 cells, chronically infected T- and myeloid-derived cell lines, as well as in fresh PBMC from 15 early-stage, asymptomatic patients [161,162]. In particular, the expression of antisense transcripts in unstimulated, HIV-1 chronically infected U1 cells was significantly higher than sense transcription, which was barely detectable [161].

In their 2007 study, Landry et al. provided more convincing evidence of antisense transcription in the HIV-1 proviral genome [163]. The authors noted that in retroviral genomes that produce transcripts in both orientations, sense transcription is more abundant than antisense transcription. They reasoned that the positive RT-PCR signal may be an artefact due to the amplification of cDNA molecules generated through endogenous priming of sense transcripts by degraded RNA or DNA fragments present within the extracted RNA pool, or through self-priming [163,164]. The occurrence of endogenous or self-priming, which yields false-positive results, can be diagnosed by conducting the reverse transcription step in the presence of the RT enzyme, but in the absence of RT primers. To avoid the possibility of endogenous and/or self-priming, Landry et al. used a strand-specific RT-PCR assay, which involves an RT primer with a 3′ half complementary to the RNA of interest and a 5′ half containing an exogenous sequence introducing a tag at the 5′ end of the cDNA. The tag provides a cDNA-specific template for one of the primers in the PCR reaction, allowing the amplification of the intended target cDNA [163]. Strand-specific RT-PCR was also used in at least two subsequent studies that confirmed antisense transcription in chronically-infected cell lines, acutely-infected primary human CD4+ T cells and resting CD4+ T cells isolated from peripheral blood of virally-suppressed HIV-1 patients [165,166]. An alternative approach to avoid endogenous priming is the use of a biotinylated RT primer and subsequent enrichment of the cDNA of interest via streptavidin beads [167]. This method was used to confirm the expression of antisense transcripts in HIV-1 infected individuals [167]. Several other studies directly or indirectly confirmed antisense transcription within the HIV-1 genome using various models [165,168,169,170,171,172,173,174,175,176].

### 4.2. Structure of the HIV-1 Natural Antisense Transcripts

The start site, length and polyadenylation site of HIV-1 antisense transcription have been the focus of several reports. The first study in this area investigated the structure of the antisense transcript through the use of a cDNA library constructed from acutely-infected A3.01 cells (Figure 1) [162]. Screening of the cDNA library yielded three clones containing HIV-derived antisense transcripts. Although two of the cDNAs were truncated, the third one, extended by 2242 bp, contained the proposed antisense ORF [158] and terminated in a poly-A tract [162]. The 5′ ends of all three cDNAs were found to map in the R region of the 3′LTR. This result was in line with a report that mapped the transcription start site at position +25 of the R region, both in in vitro transcription reactions and in transfection experiments [177]. Landry et al. performed transfection experiments, followed by 5′ RACE and 3′ RACE, and identified multiple transcription start sites in the U3 region, *nef* and *env* genes, as well as a polyadenylation signal in the *pol* gene (Figure 1) [163].

In 2012, Kobayashi-Ishihara et al. reported an in-depth analysis of HIV-1 antisense transcripts expressed after in vitro transfection of a recombinant HIV-1 molecular clone, and after infection with HIV-1_NL4–3_ [165]. This study identified four classes and subclasses of both spliced and unspliced antisense transcripts expressed in transfected cells—class I of ~10kb; class II of 5.5kb; classes III-i, III-ii and III-iii of 3-4kb; and classes IV-i and IV-ii of ~2kb. Only unspliced forms (classes I, II, III-iii and IV-ii) were also detected after infection with replication-competent HIV-1_NL4-3_ [165]. Furthermore, analysis of HIV-1_NL4-3_-infected MAGIC-5A cells, a CCR5-expressing HeLa/CD4+ cell clone 1-10, identified a major NAT (*ASP-L*) of 2574 nt with a start site at position 9451 (in the U3 region of the 3′LTR) and a termination site at position 6878 (in *env*) of the HIV-1_NL4-3_ genome (Figure 1) [165]. Similarly, strand-specific RT-PCR analysis of RNA from acutely-infected cell lines and primary human cells and chronically infected cell lines demonstrated that in all these models, the *ASP-L* start site is located within the U3 region of the 3′LTR, between residues 9441 and 9538; whereas 3′ RACE analyses determined the termination site to be located within the *env* gene, between positions 6727 and 6875 of the HIV-1_NL4-3_ genome [165]. Finally, the same study demonstrated that in acutely-infected cell lines and primary cells, and in chronically-infected cell lines > 75% of HIV-1 NATs have a predominantly nuclear localization [165]. By contrast, Saayman et al. reported that in two chronically-infected cell lines, some HIV-1 antisense transcripts extend for the majority of the proviral genome and are not polyadenylated [171].

Some of the inconsistencies in the results reported by various groups may be the consequence of different model systems and techniques. Nevertheless, there is overwhelming evidence that the HIV-1 proviral genome is transcribed in both orientations and that it produces one or more species of NAT. In addition, the evidence that the 5′ terminus of the HIV-1 antisense transcripts map within the 3′LTR demonstrates that these RNA molecules are not the product of read-through transcription initiated from downstream cellular promoters, but rather they are encoded in the proviral genome.

### 4.3. Regulation of HIV-1 Natural Antisense Transcription

The location of the 5′ terminus of the antisense transcripts suggested that their expression is directed by a negative sense promoter (NSP) within the 3′LTR (Figure 2) [162]. The activity of this promoter was found to be 3–9-fold lower than that of the HIV-1 positive sense promoter (PSP), and it was shown to be inhibited by Tat expression, possibly by directing the transcriptional machinery to the PSP [162,176]. Michael et al. used transfection experiments to map the NSP within the U3 region of the 3′LTR (positions 9460 to 9366), which contains critical NF-κB and USF binding sites, but no TATA box (Figure 2) [162]. Later, Peeters et al. identified an Sp1 binding site essential of NSP activity, especially in PMA-stimulated cells (Figure 2) [178].

Through systematic linker scanning analysis, Bentley et al. defined more precisely the regions of the 3′LTR with moderate, profound and variable effects on NSP activity [176]. The segment of the 3′LTR, with a profound effect on NSP activity, was mapped in the U3 region, between positions −58 and −183, relative to the positive sense transcription start site (U3-R boundary). This segment contains various transcription binding sites that were found to be critical for NSP activity—two Sp1 binding sites; two NF-κB binding sites; and LEF-1, Ets-1 and USF binding sites (Figure 2) [176]. This is consistent with the evidence that NSP activity is stimulated by treatment with agents that activate the NF-κB pathway, such as PMA and TNF-α [161,163,165,178]. Interestingly, disruption of the TATA box in U3 increased NSP activity by 4-fold, confirming that NSP is a TATA-less promoter, and suggesting that an initiator element (InR) is required to determine the antisense transcription start site (Figure 2) [165,176]. Indeed, the U3 region of the LTR contains at least two putative InRs between positions −61 and −47 (relative to the positive sense transcription start site), matching the consensus sequence 5′-Y-Y-**A**-N-W-Y-Y-3′ (where **A** = negative sense transcription start site; Y = C or T; N = any; W = A or T) [179]. A third InR was found in the R region at positions +19 to +26 [177].

Collectively, these studies found that HIV-1 antisense transcription is driven by a negative sense promoter located in the U3 region of the 3′LTR, is weaker than the positive sense promoter, and is inhibited by Tat. The activity of the negative sense promoter relies on both ubiquitous (Sp1, LEF-1, USF and Ets-1) and inducible (NF-κB) transcription factors. However, it lacks a TATA box, and instead utilizes an InR to determine the transcription start site.

### 4.4. The HIV-1 Natural Antisense Transcript as a Protein-Coding RNA

Following the seminal 1988 paper that proposed a new gene in the negative strand of the HIV-1 genome [158], several groups have investigated the expression, possible function and host responses against the HIV-1 antisense protein, ASP [161,169,170,173,175,177,180,181,182,183,184,185,186,187].

Our studies using chronically infected cell lines showed that during non-productive viral infection, ASP presents a sub-nuclear distribution. However, following cell stimulation and during productive viral infection, ASP translocates to the cytoplasm and onto the cell surface. Furthermore, after viral budding and release, ASP is also detectable on the surface of HIV-1 viral particles [186].

Cassan et al. showed that the *asp* ORF is conserved in the genome of most group-M HIV-1 strains (responsible for the global pandemic), but not in non-M HIV-1, HIV-2 or SIV strains [184]. The evidence that ~85% of group-M HIV-1 strains maintained the *asp* ORF (conservation of the *start* codon and avoidance of early *stop* codons) in a genomic region that is both very busy (the *asp* ORF overlaps the *env* gene and RRE) and under selective pressure (the *asp* ORF overlaps the V4 and V5 loops of *env*) requires a significant evolutionary effort [184]. In addition, a recent computational study by Nelson et al. showed that the frequency of nucleotide changes in the *asp* ORF resulting in nonsynonymous codons is significantly lower than that of changes resulting in synonymous codons [187]. This is most evident and particularly striking in the region of the *asp* ORF facing the *env* V4 loop on the opposite strand, which on the contrary is subject to a high frequency of nonsynonymous codon changes [187]. An additional interesting feature of ASP is that it does not have any known homologs, and thus it appears to have been created de novo, relatively recently when group M HIV-1 diverged from SIV_cpz_ (~100–150 years ago) [184]. Typically, viral proteins created de novo play a role in pathogenicity or spreading, which—in the case of ASP—is in line with its presence in the pandemic group M HIV-1 and its absence in endemic non-M HIV-1 groups [188,189,190]. 

Altogether, these studies suggest that ASP may play a role in the virus lifecycle. Although a few studies have provided initial evidence for such role, a complete understanding is still lacking.

### 4.5. Role of HIV-1 Antisense Transcripts in Viral Expression

In most cases, RNA molecules have one of three functions: protein-coding (mRNA), infrastructural (tRNA, rRNA and others) or regulatory (miRNA, lncRNA and others). Although the HIV-1 antisense transcript has a protein-coding function, as discussed above, there is ample evidence that its function goes beyond that.

Early studies by Rhodes and James showed that the expression of RNA molecules with sequence complementarity to the *Env* mRNA inhibited the replication of various HIV-1 strains in numerous cell systems by 50%–80% [191,192]. These studies concluded that the inhibitory effect involved the formation of an RNA–RNA duplex. Similar results were reported by other groups using antisense transcripts of various length [193,194,195], which also showed that the inhibitory activity of the antisense transcript did not require the expression of ASP [193].

Two reports by Kobayashi-Ishihara et al. provided further evidence that the HIV-1 antisense transcript inhibits HIV-1 replication. In the first study, the authors transiently transfected MAGIC-5A cells with a vector expressing the 2.6 kb *ASP-L* antisense transcript, infected the cells with HIV-1 and monitored viral replication. They showed that *ASP-L* significantly reduced the expression of HIV-1 *Gag* RNA, the levels of HIV-1 proviral DNA and viral production in the culture supernatant [165]. Consistent results were also obtained in Molt-4 T cells stably transduced with lentiviral vectors expressing *ASP-L*. Moreover, the inhibition of viral replication was greater in clones expressing higher levels of *ASP-L* [165]. Finally, the authors showed that the knockdown of *ASP-L* expression via shRNA resulted in increased viral replication [165]. The second report by Kobayashi-Ishihara et al. employed a different cell model to confirm that knocking down *ASP-L* expression leads to increased levels of HIV-1 replication [174]. Additionally, the authors generated cell clones that were latently infected with HIV-1 and stably transduced with a lentiviral vector carrying the sequence for the expression of *ASP-L*. They showed that treatment with PMA/ionomycin or vorinostat reversed HIV-1 latency in clones that failed to express *ASP-L* (despite carrying an intact lentiviral vector), but not in clones expressing the *ASP-L* RNA [174].

**Figure 3:**
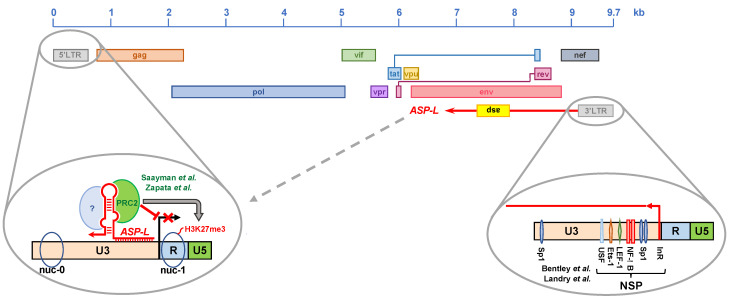
Role of HIV-1 NATs as inducers of proviral latency. Multiple reports have shown that HIV-1 NATs have a negative regulatory effect on the transcriptional activity of the HIV-1 5′LTR and contribute to the establishment and maintenance of HIV-1 latency. The suppressive activity of HIV-1 NATs has been shown to occur via epigenetic regulation of the 5′LTR, and it entails the recruitment of chromatin-modifying enzymes such as DNA methyltransferase 3a and the histone methyltransferase EZH2, which is a core component of polycomb repressor complex 2 (PRC2). All HIV-1 NATs originate in the U3 region of the 3′LTR, and thus share sequence identity with the U3 region of the 5′LTR. This allows NATs to interact with the 5′LTR via Watson–Crick and Hoogsteen base pairing.

Kevin Morris’ group was the first to propose that the HIV-1 antisense transcript acts as a lncRNA in promoting HIV-1 latency via epigenetic silencing of HIV-1 transcription [171]. In their 2014 report, Saayman et al. showed that the expression of siRNA directed against the HIV-1 antisense transcript resulted in increased levels of viral replication. Interestingly, the authors showed that the knockdown of HIV-1 antisense transcript levels was also associated with reduced levels of suppressive epigenetic marks (H3K9me2 and H3K27me3) at the 5′LTR (Figure 3). Finally, they demonstrated that the HIV-1 antisense transcript interacts with DNA methyltransferase 3a (DNMT3a) at the HIV-1 5′LTR, and found significantly lower levels of the chromatin modifying enzymes EZH2 and HDAC1 in cells expressing siRNAs directed against the HIV-1 antisense transcript [171]. Therefore, these studies provided the first evidence that an HIV-encoded NAT regulates the expression of its paired sense transcript at the epigenetic level.

Our group further investigated the role of HIV-1 antisense transcripts in epigenetic silencing of the provirus [166]. For our studies, we used the cell line Jurkat E4, which was infected with a latent HIV-1 provirus containing the GFP reporter gene [196]. Re-stimulation of HIV-1 expression with T cell activators or latency reversing agents (LRAs) can be monitored by means of GFP expression [196]. We generated a Jurkat E4-derived cell line carrying a stably transduced lentiviral vector expressing the *ASP-L* NAT described by others [165]. Point mutations in the *ASP-L* sequence prevented translation of the ASP protein, thus allowing us to evaluate the activity of the NAT in the absence of its protein product. We found that ectopic overexpression of *ASP-L* suppressed basal HIV-1 transcription during latency, and inhibited latency reversal after treatment with TNF-α or LRAs (the HDAC inhibitor SAHA and the PRC2 inhibitors EPZ-6438, EI1 and CPI-169). In addition, *ASP-L* accelerated the re-establishment of latency. Ectopic overexpression of *ASP-L* maintained high levels of PRC2 and the suppressive epigenetic mark H3K27me3 at the HIV-1 5′LTR, even after treatment with LRAs. Finally, we provided evidence that in chronically infected cell lines, naturally-expressed *ASP-L* interacts with the epigenetic silencer PRC2 (Figure 3) [196]. The ability of *ASP-L* to interact with additional transcription and epigenetic factors remains to be investigated.

Thus, there is mounting evidence that some HIV-1 NATs may act as scaffold lncRNAs that are able to recruit and tether multiple protein complexes that regulate the activity of the HIV-1 5′LTR.

## 5. Open Questions and Future Research Avenues

Although the existence of an antisense gene in the HIV-1 proviral genome was first proposed more than 30 years ago [158], many aspects of its investigation remain largely unexplored and lag far behind those of other retroviruses, such as the HTLV-1 antisense gene *hbz* [140]. Below, we attempt to address some of the questions pertaining to HIV-1 antisense transcription that remain open.

### 5.1. One or Multiple HIV-1 Antisense Transcripts?

As discussed above, different research groups have described multiple HIV-1 antisense transcripts that differ in start site, length and polyadenylation status. These differences can be explained by multiple factors, such as the cell system (primary cell vs. cell line model), the virus (replication competent vs. incompetent) and the stage of infection (acute vs. chronic vs. latent). An important factor that may also play a role is the proviral integration site, as the state of the chromatin surrounding the provirus may impact the transcriptional activity of the NSP, and consequently the structural properties of the antisense transcript. Studies involving patient samples will conclusively address the question of whether HIV-1 expresses single or multiple antisense transcripts, and—in the case of the latter—how they differ from one another.

### 5.2. Does HIV-1 Express Antisense Transcripts In Vivo?

At least two recent reports have documented antisense transcription in HIV-1-infected cells collected from donors on ART using qRT-PCR [166,167]. Zapata et al. detected *Ast* RNA levels ranging from 10 to 30 copies per million resting CD4+ T cells [166]. Mancarella et al. observed similar levels of *Ast* RNA but only following ex vivo anti-CD3/CD28 stimulation of donor CD4+ T cells [167]. Since both studies determined levels of *Ast* RNA from cDNA in bulk, further investigation is needed to determine if *Ast* expression can be detected at the single-cell level. If so, then the following questions can be examined. What fraction of HIV-1 infected cells express *Ast* and to what levels in single HIV-1-infected cells from donors on ART? Are the levels and fraction of HIV-1-infected cells with *Ast* different in donor samples from different timepoints or across donor groups (acute vs. chronic vs. elite controllers)? Is *Ast* preferentially expressed in specific cell subsets? Lastly, what is the length(s) of the antisense transcript expressed in vivo? By exploring these questions about *Ast* expression in vivo, we could gain further understandings of HIV-1 persistence in donors on ART.

### 5.3. Does Ast Affect Host Gene Expression?

LncRNAs usually act in a highly gene-specific manner, which provides a major argument in favor of exploring the use of HIV-1 antisense transcripts as a way of stabilizing proviral silencing. However, any clinical application of HIV-1 antisense transcripts via gene therapy would first require verification that they do not affect the expression of host genes. Our group has obtained preliminary evidence in support of that notion from RNA-seq studies in cell line models stably transduced with lentiviral vectors expressing *ASP-L*. This seems to indicate that at least one form of HIV-1 antisense transcript shown to promote silencing of the proviral 5′LTR [166] does not consistently affect the expression of host genes. However, these studies will need to be followed up with more formal analyses in primary cell systems and animal models.

### 5.4. Is Ast Expression Regulated at the Epigenetic Level?

The presence and position of the nucleosomes nuc-0 and nuc-1 in the HIV-1 5′LTR are well established. The HIV-1 3′LTR shares an identical sequence, and it has been shown to contain a Tat-dependent PSP and a Tat-independent NSP [176,197]. However, the role of epigenetics and chromatin remodeling on the activity of PSP and NSP in the 3′LTR remains largely unknown. Preliminary studies from our laboratory have shown that the activity of the 3′LTR NSP is under epigenetic control, and that several classes of LRAs (e.g., HDAC inhibitors, PRC2 inhibitors, DNA methyltransferase inhibitors and PKC agonists) increase antisense transcription from the 3′LTR. These results merit further investigation, because they may in part explain why LRAs have shown limited efficacy in ‘shock and kill’ cure strategies—concurrent upregulation of antisense transcripts may counteract or dampen the effect that LRAs exert on sense transcription.

### 5.5. Why Does HIV-1 Need Antisense Transcription?

As discussed above, sense–antisense transcription establishes a self-regulatory, on–off switch system that allows the fine control of gene expression. There is evidence that this may also be the case for HIV-1 [166]. Antisense transcription within the proviral genome may be advantageous to HIV-1 in several ways that would allow the virus to ‘sense’ the environment through the activation state of the cell, and to adapt its expression accordingly. First, in conditions of cell quiescence that are not conducive to viral expression, production and replication, NATs would contribute to minimizing leaky expression of HIV-1 proteins, thus reducing the chances that the infected cell may be recognized and eliminated by the immune system. Second, upon encountering an activating stimulus, NATs would set a threshold for the intranuclear levels of NF-κB and NFAT that are needed to overcome the repressive effects of NATs, to trigger the on–off switch and to achieve full transcriptional activity. When that threshold is reached, the on–off switch (from latency to viral expression) would occur rapidly rather than gradually, thus allowing HIV-1 to quickly reach maximal expression levels. Third, upon removal of the activating stimulus, NATs would allow HIV-1 to recover its basal state faster and without inducing further cell death, to establish latency and thus to persist. If this hypothesis is correct, then latency would not just be a consequence of cell quiescence that HIV-1 rides passively. On the contrary, the expression of NATs would allow HIV-1 to have some level of control over latency, and to exploit it to its own benefit.

### 5.6. Can HIV-1 NATs Be Exploited in Cure Strategies?

The therapeutic application of NATs was first proposed in the mid 2000s, mostly as drug targets [198]. Sense–antisense transcription in the HIV-1 proviral genome appears to function as a self-regulatory on–off switch [166]. We hypothesize that ectopic overexpression (10-, 100-, 1000-fold over endogenous levels) of HIV-1-derived NATs (such as *ASP-L*) represents a potential strategy to achieve a functional cure for HIV-1 in the context of ‘block and lock’ approaches. Indeed, ectopic overexpression of NATs in infected cells would contribute to silencing viral expression in three ways. First, it would further suppress the leaky expression of latent proviruses, reducing local and systemic immune activation, which contributes to cell proliferation and clonal expansion. Second, the ectopic overexpression of NATs would raise the threshold required to trigger the on–off switch, and to achieve maximal viral expression. Third, in the event that this threshold is reached, ectopic overexpression of NATs would accelerate the return to latency and reduce viral expansion. Finally, the ectopic overexpression of NATs would also be beneficial in uninfected cells, by rendering them a less ‘fertile’ environment for the expression of incoming viruses, and by increasing the likelihood that upon HIV-1 infection the integrated provirus would enter directly into latency. Altogether, a high copy number of HIV-1 derived NATs would tilt the balance toward latency, promoting deeper and more sustained proviral silencing.

### 5.7. What Would Be the Advantage of an Ast-Based Cure Strategy?

No cure strategy proposed or tested so far is perfect, and all strategies present advantages and disadvantages. It is quite possible that, ultimately, curing HIV-1 will require a combination of different approaches that are able to target subsets of infected cells in different tissues, and to act cooperatively and synergistically. The weakness in all ‘block and lock’ strategies is that they do not aim at achieving viral eradication. Rather, they seek to exploit a natural phenomenon (viral latency), to reinforce it through various strategies and possibly to make it irreversible.

In the case of a NAT-based cure strategy, an additional and important advantage is the high specificity of HIV-1-derived NATs for the provirus, thus eliminating or minimizing the chances of off-target effects. Our published studies, as well as those reported by others [166,171], indicate that HIV-1 NATs act as lncRNAs that recruit DNA- and chromatin-modifying enzymes to the HIV-1 5′LTR, promoting epigenetic changes that result in proviral silencing. The interaction of lncRNAs with their DNA targets involve Watson–Crick and Hoogsteen base pairing, which require high sequence homology. The 5′ end of HIV-1 NATs is derived from the U3 region of the 3′LTR, and thus shares perfect sequence identity with the U3 region of the 5′LTR. This perfect sequence homology would allow the interaction of NATs with the 5′LTR and the tethering of DNA- and chromatin-modifying enzymes that promote HIV-1 latency. On the other hand, HIV-1 derived NATs do not present significant sequence homology with any host genomic sequence, reducing the likelihood that they may exert an effect on any cellular gene.

### 5.8. Are NAT-Induced Epigenetic Changes Inheritable by Daughter Cells after Cell Division?

In order for the epigenetic changes induced by HIV-1 derived NATs to be curative, they must persist for the lifespan of the cell and be inherited by daughter cells after cell division. It is reasonable to hypothesize that as long as ectopic NAT overexpression persists, so do the epigenetic changes it promotes. However, the key questions are whether the NAT-dependent epigenetic modifications persist for the lifespan of the infected cell and whether they are transmitted to the daughter cells even if ectopic NAT expression decreased or is lost.

A possible answer may come from other fields of study. As discussed above, lncRNAs are responsible for the deposition of epigenetic marks on the chromatin that determine the lifelong repression of certain genes. An example is ‘genomic imprinting’, namely the silencing of one of the two parental alleles in a cluster of genes during gametogenesis [199]. Another example is ‘lyonization’, namely, the inactivation of one of the two X chromosomes in female cells that ensures X-linked gene dosage compensation [200]. In both cases, the epigenetic changes that regulate gene expression persist for the entire lifespan of the cell and are transmitted to all daughter cells. However, these mechanisms are the product of millions of years of evolution, are genetically programmed and occur in the germ line, during embryonic development or in progenitor cells.

Can inheritable epigenetic events be established in terminally differentiated somatic cells in the absence of a genetically encoded program? Evidence from several fields suggests that this may be the case. One example comes from studies showing that the long-term effects of cocaine and other drugs of abuse on neural plasticity involve alterations of histone modifications known to play a role in memory and learning [201,202]. Additional evidence comes from studies in plants in which transient changes in environmental salinity (hyperosmotic stress) result in changes in long-term somatic memory, which include epigenetic, transcriptional and physiological changes [203,204].

Are permanent, inheritable epigenetic marks possible in the case of HIV-1? The evidence that HIV-1 replication resumes following the interruption of antiretroviral therapy would suggest that permanent viral silencing is unattainable. On the other hand, intact and apparently replication-competent proviruses cannot always be reactivated ex vivo even after multiple rounds of stimulation [205]. Although this may be infrequent and occur only under special circumstances, it suggests that in some cases latency is indeed very stable. In addition, some patients are able to control viral replication through mechanisms that are still not fully understood, but which may also include more efficient and persistent epigenetic silencing. In the context of a ‘block and lock’ cure strategy, it is possible that the ectopic overexpression of HIV-1 NATs may be capable of tilting the balance between viral expression and viral latency so far in the direction of the latter that it may become irreversible and maintained after cell division. Future studies will have to determine whether ‘proviral imprinting’ is indeed achievable.

## 6. Concluding Remarks

The existence of antisense genes has been documented in many systems, viral and cellular, prokaryotic and eukaryotic, animal and vegetal. In some cases, they have been shown to produce non-protein coding transcripts, and in others to produce protein-coding transcripts. Their function has been the focus of many studies, which have shown how NATs play a central role in regulating multiple viral and cellular functions. HTLV-1—a close relative of HIV-1—is no exception, and both the *Hbz* transcript and HBZ protein have been shown to play a central role in the virus lifecycle and viral pathogenesis.

The study of the HIV-1 antisense transcript and protein, and the role that they play in viral infection and pathogenesis, lags far behind. Indeed, they are often still ignored or their expression in vivo is questioned, despite mounting evidence over three decades that points to the contrary. Further investigation into the HIV-1 antisense gene and its products—both RNA and protein products—is needed in order to gain a deeper understanding of their role in the virus lifecycle, and to determine whether they can become new tools in our arsenal against a virus that continues to kill millions of people every year throughout the world.

## Figures and Tables

**Table 1:** NATs in eukaryotic systems.

Mechanism	NAT	Organism	Function/Effects	References
Epigenetic Silencing	*Tsix*	Mammals	Recruits DNA methyltransferases to induce *Xist* promoter methylation, which inhibits its expression and prevents gene inactivation	[79,80,81,82,83]
	*ANRIL*	*H. sapiens*	Recruits PRC1 and PRC2 to induce histone methylation (H3K27) and mono-ubiquitination (H2A-K119) for silencing and repression of the *INK4* locus	[84,85,86,87]
	*Kcnq1ot1*	*H. sapiens*	Silences genes within *Kcnq1* loci on the paternal allele by recruiting chromatin modifiers, which induce repressive histone modification and DNA methylation	[88,89,90,91,92]
Transcriptional Interference	*RME2*	*S. cerevisiae*	Blocks transcriptional elongation of *IME4* transcript	[93,94]
	*SUT719*	*S. cerevisiae*	Acts as a regulatory hub linking the expression of divergent neighboring genes *GAL80* and *SUR7* and establishes a threshold-dependent on–off switch	[58,76]
RNA Stability	*BACE1-AS*	*H. sapiens*	Masks the miR485-5p binding site and prevents miRNA-mediated degradation of *BACE1* mRNA	[69,95,96]
RNA Masking	*ZEB2-AS*	*H. sapiens*	Prevents splicing of an IRES-containing intron, resulting in transcription of an alternate isoform of *ZEB2*	[78,97,98,99]

**Table 2:** NATs in viral systems.

Mechanism	NAT	Virus	Function/Effects	References
Epigenetic Silencing	LATs	Herpesvirus (HSV)	Regulates viral lytic gene expression by limiting transcripts and silencing their promoters via heterochromatinization during latency May promote latency reactivation by inhibiting apoptosis and promoting cell survival	[102,103,104,105,106,107,108,109,110,111,112,113,114,115,116,117,118,119,120,121,122,123]
	*VLT*	Varicella zoster virus (VZV)	Suppresses the expression of ORF61 to regulate latency, similar to LATs in HSV	[124]
Transcriptional Interference	*Hbz*	Human T cell leukemia virus 1 (HTLV-1)	Induces host genes involved in cell cycle progression and proliferation and anti-apoptosis factors, such as survivin May play a role in leukemogenesis with HBZ protein	[77,125,126,127,128,129,130,131,132,133,134,135,136,137,138,139,140,141,142,143,144,145,146]
Unknown	*ALT*	Kaposi’s sarcoma-associated herpesvirus (KSHV)	May play a role in regulating the viral lifecycle	[147]
